# Toward a systems-level understanding of gene regulatory, protein interaction, and metabolic networks in cyanobacteria

**DOI:** 10.3389/fgene.2014.00191

**Published:** 2014-07-02

**Authors:** Miguel A. Hernández-Prieto, Trudi A. Semeniuk, Matthias E. Futschik

**Affiliations:** ^1^Systems Biology and Bioinformatics Laboratory, IBB-CBME, University of AlgarveFaro, Portugal; ^2^Centre of Marine Sciences, University of AlgarveFaro, Portugal

**Keywords:** meta-analysis, cyanobacteria, systems biology, networks, metabolic pathways

## Abstract

Cyanobacteria are essential primary producers in marine ecosystems, playing an important role in both carbon and nitrogen cycles. In the last decade, various genome sequencing and metagenomic projects have generated large amounts of genetic data for cyanobacteria. This wealth of data provides researchers with a new basis for the study of molecular adaptation, ecology and evolution of cyanobacteria, as well as for developing biotechnological applications. It also facilitates the use of multiplex techniques, i.e., expression profiling by high-throughput technologies such as microarrays, RNA-seq, and proteomics. However, exploration and analysis of these data is challenging, and often requires advanced computational methods. Also, they need to be integrated into our existing framework of knowledge to use them to draw reliable biological conclusions. Here, systems biology provides important tools. Especially, the construction and analysis of molecular networks has emerged as a powerful systems-level framework, with which to integrate such data, and to better understand biological relevant processes in these organisms. In this review, we provide an overview of the advances and experimental approaches undertaken using multiplex data from genomic, transcriptomic, proteomic, and metabolomic studies in cyanobacteria. Furthermore, we summarize currently available web-based tools dedicated to cyanobacteria, i.e., CyanoBase, CyanoEXpress, ProPortal, Cyanorak, CyanoBIKE, and CINPER. Finally, we present a case study for the freshwater model cyanobacteria, *Synechocystis* sp. PCC6803, to show the power of meta-analysis, and the potential to extrapolate acquired knowledge to the ecologically important marine cyanobacteria genus, *Prochlorococcus*.

## Introduction

Cyanobacteria have a unique position in the living world, as they are the only prokaryotes capable of performing oxygenic photosynthesis. This confers on them unique roles in the carbon and nitrogen cycles on this planet, as well as an important primary ecological function. They thrive in diverse habitats, ranging from desert crusts to open sea (Partensky et al., 1999; Garcia-Pichel and Pringault, 2001; Garcia-Pichel et al., 2003).

Ecologically, marine cyanobacteria are at the bottom of the food pyramid and, thus, provide organic matter directly or indirectly to virtually every other marine organism (Falkowski, 2012). Specifically, marine cyanobacteria play a role of global importance, accounting for approximately 25% of the total carbon fixation in oceans through their photosynthetic activity (Flombaum et al., 2013); this is a remarkable quota, considering that approximately half of the atmospheric carbon is fixed in oceans (Field et al., 1998). The main part of this budget is fixed by planktonic picocyanobacteria, which are the most abundant photosynthetic organisms on earth (Li et al., 1983). Their most prominent representatives are from the genera *Prochlorococcus* and *Synechococcus*, which occur mainly in the euphotic zone of tropical and subtropical oceans (Flombaum et al., 2013). Notably, *Prochlorococcus* is the smallest known photosynthetic organism, ranging from 0.6 to 1 μm in diameter. Its abundance in oligotrophic marine areas reaches up to a million cells per milliliter, making them a key component of the global carbon cycle (Chisholm et al., 1988). Other planktonic marine genera, such as *Trichodesmium* and *Crocosphaera*, play a key role in nitrogen fixation in oceans (Duce et al., 2008; Langlois et al., 2008). Finally, marine benthic cyanobacteria are less abundant and have a smaller impact on the global carbon and nitrogen cycle, but are more phylogenetically diverse than the planktonic cyanobacteria. Representative examples of nitrogen-fixing marine benthic genera are the heterocyst-forming *Calothrix* and *Scytonema*, the non-heterocyst forming *Lyngbya*, *Microcoleus*, *Phormidium*, *Schizothrix* (Hoffmann, 1999), and the unicellular *Cyanothece* (Reddy et al., 1993).

Besides their global ecological importance, cyanobacteria have attracted the interest of researchers for the study of photosynthesis (Sun et al., 1999; Nelson, 2011). Several characteristics, such as their faster life cycle, simple nutrient requirements, smaller genomes, and in some cases, the ease with which genetic manipulation can be carried out (Frigaard et al., 2004; Heidorn et al., 2011; Ruffing, 2011), make them the preferred photosynthetic organism for laboratory studies. In addition to this basic research, cyanobacteria are the focus of biotechnological applications (Abed et al., 2009), especially related to energy production (Wang et al., 2012).

Their capacity for photosynthesis, along with their potential for valuable product production and bioremediation—in both ecological and technological contexts—has motivated intense research on their genomes, as a first step toward understanding these organisms. Their small genome size together with both the development and decreasing cost of new sequencing technologies have facilitated the annotation of a wide range of cyanobacterial genomes (Nakao et al., 2010; Shih et al., 2013). This plethora of genomes confers scientists with a great resource for comparative analysis (Kettler et al., 2007; Dufresne et al., 2008; Stanley et al., 2013), which is further fueled by the ongoing discovery of new genes, functions, and applications (reviewed in Scanlan et al., 2009; Partensky and Garczarek, 2010). It should be noted, however, that data generated, using these new genomic techniques, do not equally cover different cyanobacteria species. Historic biases toward model organisms, as well as the difficulty in culturing many free-living species, have both resulted in great quantities of data for specific organisms, and under-representation of others. Thus, in spite of the abundance of genomes, the vast majority of downstream studies have focused on relatively few genera, including *Synechocystis*, *Prochlorococcus*, *Synechococcus*, *Anabaena*, and *Nostoc* (Table S1).

However, the existence of a core set of genes, present in all sequenced cyanobacteria (Shi and Falkowski, 2008), might permit extrapolation from well-studied species to newly sequenced organisms, using systems-level approaches (Albert, 2007). In this context, tools from systems biology can integrate existing knowledge acquired from phylogenetically-related model organisms, like freshwater *Synechocystis* sp. PCC6803 (hereafter *Synechocystis* 6803), with new data from recently sequenced marine cyanobacteria. For example, genome comparison studies, together with *in silico* models of molecular networks provide researchers with a powerful framework to build new knowledge into, without starting from scratch for each new species. Similarly, network representations of relationships between genes (leading to regulatory networks), proteins (interaction networks), and metabolites (metabolic pathways) are intuitive systems-level tools to integrate and consolidate data from different studies, or even different organisms; such networks have been successfully applied to many diverse biological groups to date. While networks constructed directly from medium to large-scale experimental data are viewed with greater confidence than those obtained through computational extrapolation of reference networks, the later approach can still provide important supplementary information for less well-studied species, as is the case for many of the marine cyanobacteria. Thus, system-tools can provide an attractive framework for research into marine cyanobacteria.

## “omic” data: an overview of the impact of new technologies

We can date the beginning of the “omic” era in cyanobacteria to the sequencing of the first cyanobacterium genome, i.e., freshwater cyanobacterium *Synechocystis* 6803 (Kaneko et al., 1996). Its early sequencing was in part due to the relative small genome size of *Synechocystis* 6803, with a chromosome length of 3573 kb (47.72% GC content), plus 383 kb distributed between seven megaplasmids (http://genome.microbedb.jp/cyanobase/Synechocystis); and in part to its established presence as a model organism in many laboratories (e.g., there are 431 entries for *Synechocystis* 6803 in PubMed Central between 1950 and 1995). Notably, the availability of its genome further enhanced a bias in the literature toward *Synechocystis* 6803, making it a preferred organism for biotechnological studies, illustrated by the fact that there are almost twice as many PubMed Central records from 1996 to 2013 for *Synechocystis* 6803 than for the next most cited cyanobacterium genus *Anabaena* (2623 compared with 1580 for *Anabaena*). This bias in the literature is remarkable given that the completely sequenced genome of *Anabaena* sp. PCC 7120 was published in 2001 (Kaneko et al., 2001).

**Figure 1:**
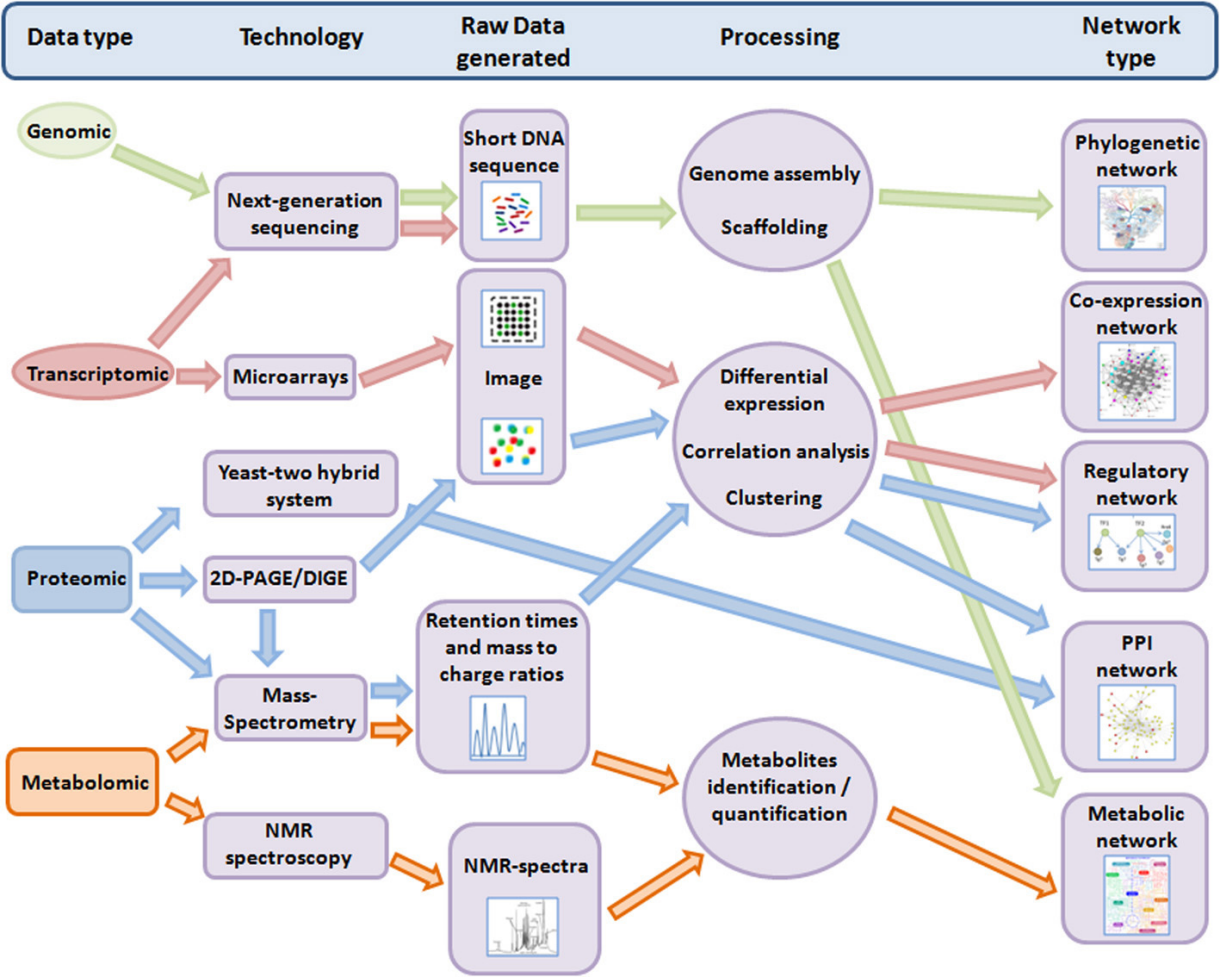
**Overview of the workflow from different “omic” methods to different systems-level networks**. Only technologies discussed in this review are shown. For more details, the manually-curated meta-database OMICtools (http://omictools.com/) provides tools and platforms for multi-omic data analysis. Note: 2-D PAGE, two-dimensional polyacrilamide gels; DIGE, difference gel electrophoresis; NMR, nuclear magnetic resonance; PPI, protein-protein interaction.

Nevertheless, in the last decade, advances in high-throughput techniques (HTT) for genetic analysis have compounded the data available for marine and other cyanobacteria on genomic, transcriptomic, proteomic and metabolomic levels (Ow and Wright, 2009). Importantly, this technological revolution in “omic” data has paved the way to the development of methods to collate and integrate such data into a systems-level framework (Figure 1). We highlight some of the major advancements in these fields for cyanobacteria—with a focus on marine cyanobacteria—below.

## From genomics to metagenomics: from the ocean to the laboratory and back to the ocean

**Figure 2:**
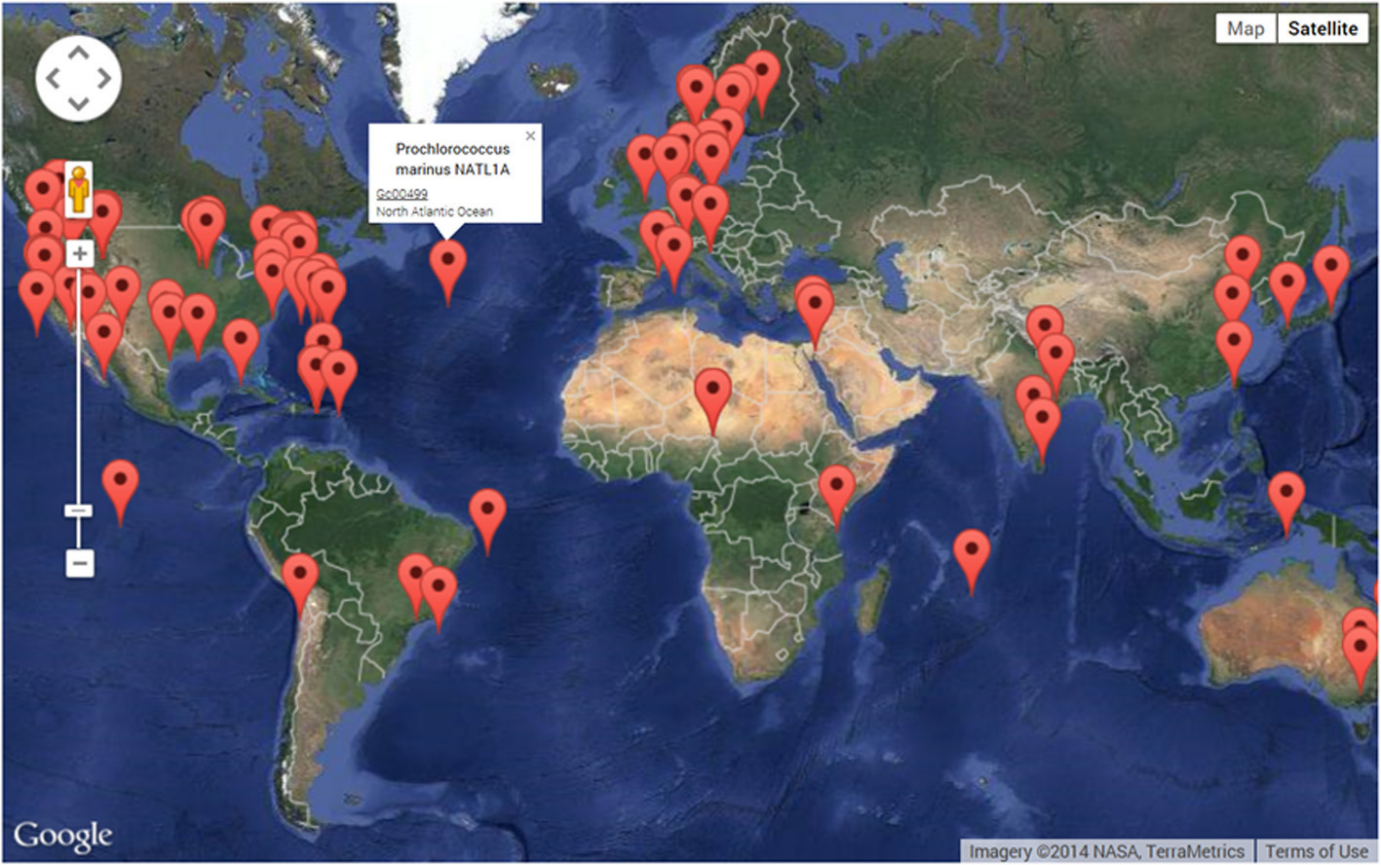
**World map showing genomic records for cyanobacteria generated using an interface powered by google maps, available on the Genomes Online Database (GOLD) website (http://genomesonline.org/cgi-bin/GOLD/index.cgi)**. Red labels indicate the original location of a specifically sequenced strain. Labels direct the user to information on the organism, genome characteristics (i.e., GC content, size), sequencing method used, specific coordinates of the origin of the strain, as well as links to external databases (as shown for *Prochlorococcus marinus* NATL1A). The GOLD also describes the status of each record in tabular format.

Assemblage of the genome sequences of members of the two most abundant marine cyanobacteria genera took place almost a decade later than for *Synechocystis* 6803. In Dufresne et al. (2003) presented the genome of the low-light adapted strain *Prochlorococcus marinus* SS120, while Rocap et al. (2003) published the genome of the high-light adapted strains *Prochlorococcus marinus* MED4 and MIT9313; in the same issue as Rocap et al. (2003), the genome sequence of *Synechococcus* sp. WH8102 was described by Palenik et al. (2003). More recently, genomic data acquisition has been driven by the development of so-called next generation sequencing techniques (to differentiate them from the sequencing technique, originally developed by Sanger) and a drastic decrease in cost (Liu et al., 2012). Currently, data are generated in the pursuit of scientific and biotechnological objectives in multiple species-specific genome projects, as well as in global metagenomic projects, in which cyanobacteria are also identified (Figure 2). The plethora of data resulting from these projects is commonly available through public repositories for the benefit of the scientific community. Genomic data for cyanobacteria are accessible through several general as well as organism-specific repositories (Table 1). Specific repositories include CyanoBase (Nakamura et al., 1998) with 39 cyanobacterial genomes available, as well as repositories focussed exclusively on marine picocyanobacteria, i.e., Cyanorak (Dufresne et al., 2008; Scanlan et al., 2009) and ProPortal (Kelly et al., 2012). While more general repositories are the integrated microbial genomes (IMG) database from the Department of Energy (DOE, USA) (Markowitz et al., 2012) with 89 complete cyanobacterial genomes, and the National Center for Biotechnological Information (NCBI) database with more than 100 cyanobacterial genome sequences available. A great part (54) of the genomes included in NCBI database resulted from the CyanoGEBA project (Shih et al., 2013), which aims to enhance the phylogenetic diversity available in public repositories by providing information on cyanobacterial taxa that were previously under-represented (Table S1). In addition to laboratory-based efforts, global-scale metagenomic projects, such as the Global Ocean Survey (Rusch et al., 2007), have sequenced populations of marine microorganisms collected around the globe, and vastly extended the number of sequences available to the research community and industry (reviewed in Lorenz and Eck, 2005). For instance, metagenomic data for marine habitats are available through CAMERA (http://camera.calit2.net/). Thus, our research efforts over the last decade have significantly expanded our knowledge of cyanobacteria compared with the status for earlier reviews in this field (e.g., Burja et al., 2003).

**Table 1:** **Publically available full genome sequences for cyanobacteria in various repositories as at April 2014**.

**Name**	**Type**	**No. full genomes**	***^#^P* records**	^*^S records	**Weblink**
Cyanorak	MC	14	3	11	http://www.sb-roscoff.fr/cyanorak
ProPortal	MC	68 (44 phages)	13	11	http://proportal.mit.edu/
CyanoBase	C	39	12	10	http://genome.microbedb.jp/cyanobase/
CyanoBIKE	C	27	11	12	http://biobike-8003.csbc.vcu.edu/biologin
NCBI	G	103	12	13	http://www.ncbi.nlm.nih.gov/
IMG (JGI)	G	208	17	15	http://img.jgi.doe.gov
KEGG	G	75	12	13	http://www.genome.jp/kegg/
Microbes online	G	50	17	12	http://www.microbesonline.org/
SEED	G	161	28	35	http://pubseed.theseed.org/

# Prochlorococcus;

* Synechococcus;

MC, marine cyanobacteria; C, cyanobacteria; G, general.

Clearly, the public availability of genome sequences for different cyanobacteria has had considerable impact on research directions. It helped laboratory-based researchers to direct molecular and biochemical work toward specific genes, e.g., toward those identified as novel, or toward those conserved in other organisms. For instance, new genome sequences can be exploited to identify “orphan pathways,” in which metabolites were previously detected, but not the gene clusters responsible for their biosynthesis (Gross, 2007). Indeed, this was the case for the pathway responsible for the biosynthesis of patellamides (didemnid peptides with potential medical applications) (Ireland et al., 1982; Williams and Jacobs, 1993). This metabolite was initially detected in the symbiotic cyanobacteria *Prochloron didemni* (Degnan et al., 1989), and later in the bloom-forming cyanobacterium *Trichodesmium erythraeum* IMS101. Sequencing of the gene set responsible for its synthesis in *P. didemni* and function confirmation through cloning in *Escherichia coli* facilitated the identification of the counterpart gene cluster, based on similarity, in *T. erythraeum* IMS101 (Schmidt et al., 2005).

Unfortunately, newly identified functions of genes or proteins do not necessarily result in the update of annotations in genome repositories, which typically lag behind our current knowledge. To address this problem, a community-database named CYORF (http://cyano.genome.ad.jp/) was set up to annotate newly described functions with their corresponding genes, and to allow scientists to actively curate these annotations. Just the initial effort within the Japanese scientific community resulted in about 1000 gene function re-annotations (Furumichi et al., 2002). This database has been superseded by a social genome annotation tool called TogoAnnotation (http://togo.annotation.jp/) open to the entire scientific community (Fujisawa et al., 2014).

**Figure 3:**
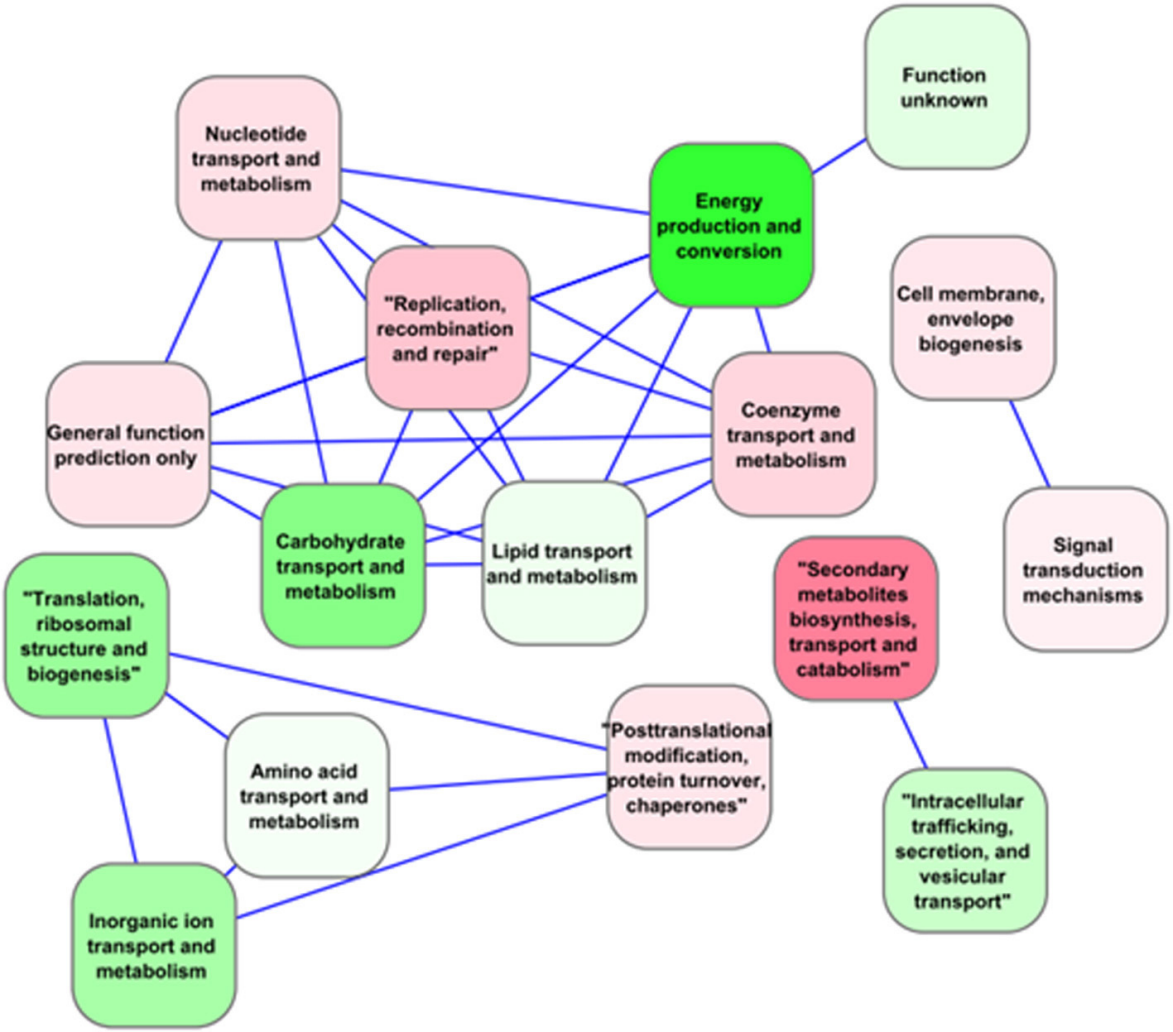
**Correlation network for functional categories (as defined by Falkowski, 2012) based on the expression of “core” genes in *Synechocystis* sp. PCC 6803 under multiple environmental conditions extracted from CyanoEXpress**. Nodes represent Gene Ontology (GO) pathways, colored based on their average differential expression in the dark such that gradients of red indicate induction, while green indicate repression. Only categories with an absolute Spearman correlation value greater than 0.95 were connected by an edge.

Despite public accessibility to genomic data, only few comparative studies have been carried out to date (Scanlan et al., 2009). For example, a comparison of *Prochlorococcus* genomes distinguished between “core” genes present in all, and “flexible” genes that were not conserved in all the branches of the phylogenetic tree (Kettler et al., 2007). This approach was expanded to include other cyanobacteria species, including fresh-water types, reducing the number of core genes from 1273 to 323. These core genes are significantly enriched in key photosynthetic (12%) and ribosomal proteins (7%) over other functional categories (Shi and Falkowski, 2008) (Figure 3). The presence of core and flexible genes also served to estimate the importance and relative contribution of vertical inheritance vs. horizontal gene transfer for each of these gene fractions in 11 *Synechococcus* strains. The estimated number of gene families present in the core genome of these *Synechococcus* strains was 1572, adding three *Prochlorococcus* strains to this comparative analysis reduced the number to 1228 gene families. The number of unique genes varied greatly between strains, from 91 to 845 genes, and was correlated with genome size. Two *Synechococcus* subclusters WH5701 and RCC307 were an exception, since they presented a higher number of unique genes than expected for their genome size. The presence of these unique genes in genomic islands and their horizontal transfer likely confers advantages for adaption to narrow ecological niches (Dufresne et al., 2008). Avrani et al. (2011) also reinforced the importance of genomic variability in adaptation of natural populations. Their work revealed the importance of genomic variability within a bacterial community for viral resistance. Starting with four high-light adapted *Prochlorococcus* strains, 77 sub-strains resistant to infection by 10 different podoviruses were selected to further characterize the genomic region responsible for this resistance. Using next-generation sequencing technologies, 27 resistant and eight control sub-strains were sequenced to identify resistance specific mutations. Other sub-strains were screened by sequencing of PCR amplicons. These results demonstrated that phage infection promotes enhanced diversity. It also revealed the importance of genomic islands (regions in the genome acquired by horizontally transfer) in phage resistance, since most mutations accumulated in these regions. The preferential location of viral-attachment genes in genomic islands imposes a selective pressure on genomic islands, and island gene-content diversity. These findings have direct implications on our understanding of the ecology of *Prochlorococcus* communities and of the mechanisms supporting the long-term coexistence of this cyanobacterium with its phages (Avrani et al., 2011).

Data from metagenomic studies have been very useful for clarification of the genetic content of cyanobacteria. Sampling and carrying out genomic DNA isolation directly from a particular environmental niche circumvents problems related to the difficulty in culturing many of these organisms. Such was the case for the unicellular N2-fixing UCYN-A cyanobacteria, where attempts to cultivate have so far failed. UCYN-A nitrogenase genes are maximally expressed during the light period (Church et al., 2005; Zehr et al., 2007), contrary to the expression pattern observed for *Cyanothece* nitrogenase genes, which separate temporally oxygen evolution from nitrogen fixation to avoid inhibitory effects (Stockel et al., 2008; Toepel et al., 2008; Welsh et al., 2008). The use of enriched fractions obtained by fluorescence-activated cell sorting helped to overcome the difficulty to separate UCYN-A cells from other small phototrophic and heterotrophic populations using traditional flow cytometry. Metagenomic analysis of this enriched fraction resulted in at least a 10-fold genomic coverage of the UCYN-A genome. Comparison of these results with the cyanobacterial core genome indicated that at least 79% of the core genes, as well as the nitrogenase genes were present in the UCYN-A genome. Strikingly, no UCYN-A sequences encoding proteins involved in CO2 concentration, CO2 fixation or Photosystem II were detected. The absence of Photosystem II and thus, of light-driven oxygen evolution, can explain the expression pattern of the nitrogenase genes in this strain (Zehr et al., 2008). Flow cytometry-based sorting was also used to enrich marine samples with cyanobacteria of the genus *Synechococcus*. This enrichment facilitated the assembly of contigs and the identification of at least three distinct plasmids lacking in genomes from model strains. The data obtained from a natural population showed a great genomic diversity compared with model *Synechococcus* strains isolated from the same environment, stressing the importance of horizontal gene transfer in natural populations (Palenik et al., 2009).

In general, genomic data from free-living organisms give researchers a much wider view of their molecular and ecological diversity, as well as their population dynamics. For example, the number of *Prochlorococcus* species estimated from ocean metagenomic data is in the order of 35 (Thompson et al., 2013). A good example of how metagenomic data from the global ocean survey collection have been exploited, is in the search for specific gene families. This was the case for phosphate acquisition (Martiny et al., 2009a) and nitrate/nitrite assimilation genes (Martiny et al., 2009b). Regarding phosphate acquisition, the authors established a correlation between ortho-phosphate availability in different oceanic regions and the presence or absence of phosphate uptake genes (Martiny et al., 2009a). A similar approach was taken in the later study to locate genes involved in nitrate/nitrite assimilation. Sequences related to nitrogen assimilation were sorted phylogenetically, based on their characteristic GC content and on detected homology (using both blastx and blastn) of their corresponding paired-end sequences. This distinction allowed the authors to assign sequences to specific clades or species. Importantly, this study served to establish the existence of *Prochlorococcus* strains, harboring genes that encode transporters and reductases for both nitrite and nitrate, and thus, capable of capturing and using these forms of nitrogen. This is a remarkable finding; since it was previously thought that genes for nitrate assimilation were absent in *Prochlorococcus* strains, based on the available genome sequences from cultured strains. It also indicates that generalizations based only on sequenced genomes from cultured strains should be treated with caution.

Despite the great potential of metagenomic data, identifying novel molecular mechanisms remains difficult due to inherent ambiguities of sequence assembly. For instance, the work undertaken by two research groups, using metagenomic data obtained from high-nutrient low-chlorophyll oceanic regions, revealed new clades of high-light adapted *Prochlorococcus*, lacking culture representatives. The identification of these clades was based on phylogenetic analysis of “core” functional genomes (Rusch et al., 2010), or through identification of 16S rRNA sequences (West et al., 2011). Due to assembly problems, these studies did not describe any previously unidentified gene. To circumvent this problem, Malmstrom and co-workers combined analysis of metagenomic data with single cell genomics of selected strains. Their analysis of just 10 single cells identified 394 genes, not previously described in *Prochlorococcus* strains (Malmstrom et al., 2013). In particular, they characterized genes encoding new siderophore-mediated iron scavenging mechanisms employed by *Prochlorococcus*. These genes represent an adaptation to the low iron concentrations in the ocean region, where these sequences were obtained. Curiously, the genes involved in siderophore transport are located within a genomic island, indicating that their origin was through horizontal transfer, probably mediated by phages (Kettler et al., 2007; Lindell et al., 2007).

Metagenomic data have also been used to predict functional relationships between conserved protein domains with unknown function, and those participating in a particular cellular function. This approach assumes that functional domains are only retained in the genome, when their presence gives an organism some competitive advantage in a particular environment. Under this premise, if the presence of an unknown protein domain correlates with that of domains of known function, it is inferred that it is involved in a same function; this principle of inference is commonly known as “guilt-by-association.” Formally, it can be treated using graphs, where functional domains or genes are represented by nodes. The presence of two nodes in similar niches (high correlation) is represented by an edge. The graphical association of unknown domains with those related to a particular function is taken as indicative of their functional role (Buttigieg et al., 2013). This “guilt-by-association” principle is also used in gene functional prediction based on clustering of RNA expression data, as discussed in the section on web-based tools.

## Transcriptomics: gene expression profiling from microarrays to RNAseq

A great advantage of having the full genome sequence is that it facilitates the development of comprehensive microarrays for transcriptome profiling. Thus, it is not surprising that the early publication of the *Synechocystis* 6803 genome resulted in the advent of the first whole genome microarray platform for a cyanobacterium, denoted as cyanoCHIP, and commercialized by Takara Bio. The first articles using this platform were published in 2001, dealing with changes in gene expression, as a result of changes in light intensity (Hihara et al., 2001) and temperature (Suzuki et al., 2001). Other works soon followed these initial experiments in the field of whole genome transcriptomics, and a number of alternative microarray platforms for *Synechocystis* 6803 were developed using different approaches (Postier et al., 2003; Singh et al., 2008; Zhang et al., 2008; Georg et al., 2009; Dickson et al., 2012). To date, more than 700 microarray experiments have been contributed to three main public repositories: ArrayExpress (Rustici et al., 2013), Kyoto Encyclopedia of Genes and Genomes (KEGG) Expression (Goto et al., 2000) and the Gene Expression Omnibus (GEO) database (Edgar et al., 2002). Such data contain valuable information on gene regulation under multiple environmental conditions, and genetic backgrounds. They are essential to the compilation of robust regulatory networks, using systems-level approaches (Figure 1).

**Figure 4:**
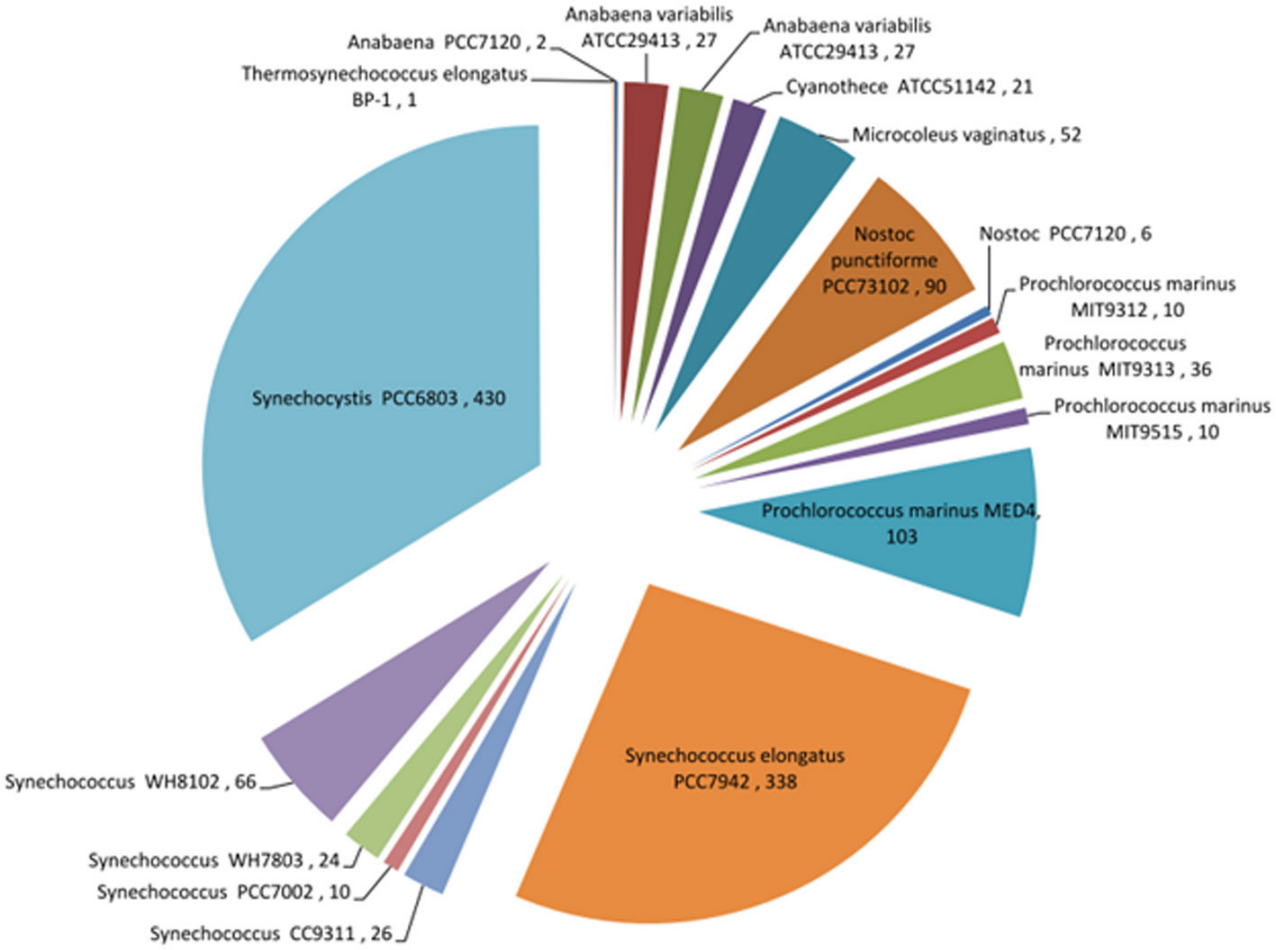
**Pie chart showing records of expression data for different cyanobacteria that are currently available in the Gene Expression Omnibus (GEO) database (http://www.ncbi.nlm.nih.gov/geo/)**. The bias toward *Synechocystis* 6803 can be clearly seen in this species breakdown of transcriptomic data. Data from April 2014.

Despite the bias toward *Synechocystis* 6803, abundant transcriptomics data also exist for other cyanobacteria (Figure 4). In the study of marine cyanobacteria, genome-level microarray analyses have frequently focused on limiting growth factors in oceans, such as iron (Thompson et al., 2011), nickel (Dupont et al., 2012), copper (Stuart et al., 2009), phosphate (Tetu et al., 2009; Ostrowski et al., 2010), and nitrogen (Su et al., 2006; Tolonen et al., 2006). Furthermore, studies were carried out to assess the effect of UV on the cell cycle (Kolowrat et al., 2010), the protective capacity to reactive oxygen species of cells acclimated to high- or low-intensity light (Blot et al., 2011), and to compare the transcriptome structures of the *Prochlorococcus* strains MED4 and MIT9313 (Voigt et al., 2014).

The earliest genome-level transcriptomic study carried out on marine cyanobacteria was on nitrogen metabolism. Su and co-workers predicted genes controlled by the global regulator NtcA of nitrogen metabolism in *Synechococcus* sp. WH8102 based on comparative genomics analysis, and mining of experimental data from related organisms (Su et al., 2005, 2006). To validate their predictions, and to incorporate specific data for *Synechococcus* sp. WH8102, they profiled RNA extracted from cultures grown on nitrate and ammonium, as the sole nitrogen source. As a result, they compiled an extended regulatory network, constituting 429 genes, of which 338 were differentially expressed in the microarray data (Su et al., 2006). Elements of this network were also found in *Prochlorococcus*. Here, microarray data served to compare two distinct ecotypes, MED4 and MIT9313, and to identify the genes differentially regulated during nitrogen stress (Tolonen et al., 2006). Temporal expression profiling revealed a faster and shorter transcriptional response for MED4 than for MIT9313. The authors argued that such differences reflected their niche-specific requirements (Moore et al., 1998; West et al., 2001; Johnson et al., 2006). The MED4 ecotype occupies the top layers of the water column, where nitrogen sources (mainly ammonium and urea) fluctuate due to high-mixing levels, and the amount of incident light is higher than for deeper layers occupied by the low-light adapted MIT9313. A faster transcriptional response in MED4 would therefore avoid damage to their photosystems under nitrogen-limited conditions (Tolonen et al., 2006). In conclusion, their respective depth distributions have resulted in different adaptations not only to light, but also to nitrogen levels.

A large amount of work has been carried out to characterize the regulatory network underlying the circadian rhythm in both planktonic and benthic marine cyanobacteria (Zinser et al., 2009; Yang et al., 2010; Axmann et al., 2014). A tight circadian expression regulation can help photosynthetic organisms to optimize cellular processes through anticipating and synchronizing transcription of photosynthetic-related genes with daylight hours. In the case of the unicellular *Cyanothece* sp. ATCC 51142, this regulation ensures that incompatible processes, such as oxygen-sensitive nitrogen fixation and evolution of oxygen through photosynthesis, do not occur at the same time (Reddy et al., 1993). Expression profiling of this *Cyanothece* during dark-light cycles showed cyclic regulation, with different clusters of genes peaking in dark or light conditions (Stockel et al., 2008; Toepel et al., 2008). The profiling served as a basis to model the regulatory network underlying this cyclic expression pattern, inferring regulatory influences for gene clusters. To do this, the authors employed a previously developed algorithm (the Inferelator) for derivation of gene regulatory networks using observed expression data (Bonneau et al., 2006). The algorithm predicts models for the expression of a single gene or cluster of genes, as a function of the expression levels of transcription factors (TFs), environmental conditions, and interactions between these factors. The model was used to infer potential key TFs in gene cluster regulation and to predict the behavior of co-expressed clusters under conditions not been used for calibration (McDermott et al., 2011). A comparable cyclic regulation was also identified in *Prochlorococcus* MED4 through comparison of mRNAs and protein expression levels of 312 genes (Waldbauer et al., 2012).

Although microarrays are powerful tools to capture transcriptional activity on a comprehensive scale, small sample size, insufficient control of false positives, and poor method description can result in findings with limited reproducibility (Ntzani and Ioannidis, 2003; Jafari and Azuaje, 2006). To overcome such limitations, the statistical combination of multiple studies (or meta-analysis) is used to enhance the significance and validity of new findings. Such meta-analyses have great potential, as studies in various other biological fields have demonstrated (Steele and Tucker, 2008; Sun et al., 2009; Genini et al., 2011). Intriguingly, only two studies have systematically combined available gene expression data for cyanobacteria to obtain a systems level overview. The first study integrated 163 microarray data sets generated using *Synechocystis* 6803 as the model organism, with genetic, as well as environmental perturbations (Singh et al., 2010). They concluded that up to 12% of the genes in *Synechocystis* 6803 responded similarly to different perturbations, indicative of a similar regulatory program. A Bayesian network, connecting different KEGG pathways based on gene expression correlation between set of genes and genes with regulatory genes, was generated. Using this network, the authors proposed a functional connection between regulatory elements that control nitrogen and carbon metabolism.

Independently, our group collated all available raw microarray data for *Synechocystis* 6803 and pre-processed them in a standardized manner. We chose to re-analyze all experiments to minimize variations in expression levels due to the use of different normalization methods by independent researchers. Our dataset currently contains more than 700 microarray measurements, and is regularly updated with newly available data. These expression profiles were clustered and made available to researchers through CyanoEXpress (http://cyanoexpress.sysbiolab.eu/), a web-based user-friendly visualization tool, for exploration of expression profiles (Hernandez-Prieto and Futschik, 2012). Gene-directed search enables rapid detection of co-expression patterns, which can be highly indicative for the annotation of less-studied genes, and in the prediction of new gene functions.

As an alternative to microarray analyses, which are based on hybridization of cDNA or mRNA in probes attached to a solid surface, the development of cost-effective and efficient next generation sequencing technologies has promoted the direct sequencing of RNA for expression profiling (Flaherty et al., 2011; Mitschke et al., 2011; Vijayan et al., 2011; Ludwig and Bryant, 2012; Ruffing, 2013). Together with the use of tiling arrays, this approach (commonly referred to as RNA-Seq) has revealed the existence of numerous non-protein coding transcripts (ncRNAs) in cyanobacteria. Previously, these ncRNAs remained undetected in microarrays, which only comprised probes for protein-coding genes. Thus, it was surprising when more comprehensive profiling approaches revealed that ncRNAs can make up a substantial part of the transcriptome in cyanobacteria. For instance, RNA-Seq data for *Prochlorococcus* indicate that up to three quarters of the genes have an antisense RNA (ncRNA transcribed from the opposed strand to a protein encoding gene) (Waldbauer et al., 2012; Voigt et al., 2014). While the detection of new ncRNA has been facilitated through RNA-Seq, their functional characterization poses a major task for the future (Haas et al., 2012). Interestingly, ncRNAs seem also to contribute to a substantial part of the sequences detected by meta-transcriptomics analyses of RNA from natural environments (Voigt et al., 2014). In fact, comparison of four meta-transcriptomic studies carried out in oceans showed that approximately 25% of the detected RNA sequences could not be assigned to protein-coding genes or rRNAs; approximately 16% of these sequences displayed structural similarity to sRNA families represented in the Rfam database (http://rfam.xfam.org/) (Shi et al., 2009; Gardner et al., 2011).

Hence, RNA-Seq has highlighted the importance of ncRNAs in cyanobacteria and provided an important method for characterizing transcription start sites. Two major drawbacks of RNA-Seq analyses compared with microarray analyses are the limited number of tools available for downstream analyses, and the larger data files, which require more powerful computers for their analysis. A further typical feature of RNA-Seq is that a small number of highly expressed genes can make up the vast majority of sequence data, hindering the detection of many lowly expressed genes. For instance, it is often necessary to remove rRNA making up the vast majority of total RNA prior to sequencing. Also, a sufficiently large number of reads need to be generated to obtain a faithful profile of the transcriptome (Haas et al., 2012). Nevertheless, RNA-Seq is rapidly becoming the standard method for cyanobacterial transcriptomics.

## Proteomics: the challenge to provide genome-level protein profiling

The lack of knowledge about post-transcriptional and post-translational regulatory processes makes it difficult to extrapolate from a gene's transcript level to the activity level of its corresponding protein. This lack of understanding of the post-transcriptional regulatory process in cyanobacteria was patent when RNA and protein levels were monitored in parallel during the diel-cycle in *Cyanothece* (Aryal et al., 2011; Stockel et al., 2011) and *Prochlorococcus* MED4 (Waldbauer et al., 2012). Whole-cell proteomics seeks to overcome this limitation by directly studying protein structures and functions, their expression levels at different cellular stages, as well as their protein-protein interactions; these aspects are essential to understand biological processes in cells (Figure 1).

Traditionally, two-dimensional polyacrilamide gels (2D-PAGE) combined with the use of different fluorescence dyes (difference gel electrophoresis; 2D-DIGE) were conceived to estimate concentration differences for each protein between two different physiological states in a similar manner to two color microarrays (Arruda et al., 2011). More recent strategies take advantage of the sensitivity of liquid chromatography (LC), coupled with tandem mass spectrometry (MS), known as LC-MS/MS, for quantitative proteomic analysis, using different tags (as reviewed in Rotilio et al., 2012).

In marine cyanobacteria, global protein studies were carried out to evaluate organism response to stress, or their variability with respect to ecotype. The most recent studies on marine cyanobacteria looked at the effects of phosphate, cadmium and zinc stress using LC-MS/MS technology on *Synechococcus* WH8102 (Cox and Saito, 2013), or nitrogen regimes using 2-D/MALDI-TOF-MS on *T. erythraeum* IMS101 (Sandh et al., 2011). The genome shrinkage process, which was identified for *Prochlorococcus* strains (Dufresne et al., 2005), makes the study of the proteome of different ecotypes in this genera of special interest to detect niche specific adaptations. Fuszard et al. (2012) investigated changes in protein levels induced upon phosphate depletion in three *Prochlorococcus* strains representing the high-light (MIT9312) and low-light ecotypes (NATL2A and SS120). Protein levels and growth rate changes were larger in the high-light ecotype than in the two strains belonging to the low-light ecotype. These results confirmed previous differences observed through genome comparisons, reflecting niche-driven molecular diversity, not only at specific gene divergences, but also at the codon usage level between different ecotypes (Paul et al., 2010).

For proteins with undefined functions, protein-protein interaction studies can assist researchers to determine their roles through their interaction with proteins (or protein complexes) with known functions (Prommeenate et al., 2004; Yao et al., 2007; Nixon et al., 2010). With this purpose in mind, *Synechocystis* 6803 protein extracts under native conditions (BN-PAGE) were separated by splitting the gel into small slices, and identifying the proteins in each slice by LC-MS/MS (Tanaka et al., 2010). These data were used to compile the protein co-migration database (PCoM-DB; http://pcomdb.lowtem.hokudai.ac.jp/proteins/top). In this way, the authors identified proteins of unknown function co-migrating with known proteins or protein-complexes.

In addition to experiments directed toward specific proteins, large-scale protein-protein interactions are crucial to post-genomic systems biology. HTT applied to proteomics, such as the yeast two-hybrid system, assisted scientists in different fields in the construction of large protein-protein interaction networks (Wallach et al., 2013; Ngounou Wetie et al., 2014). Networks generated from these studies serve to identify potential targets for future biochemical and bioinformatics studies (Kaçar and Gaucher, 2013; Yu et al., 2013). To fill this gap in the field of cyanobacteria, Sato et al. (2007) undertook the first systematic identification of protein interactions in *Synechocystis 6803*. Using yeast two-hybrid assays, they screened 1825 genes, discovering 3236 independent two-hybrid interactions (Sato et al., 2007). Such interaction data are important for functional analyses of genes in *Synechocystis* 6803, as well as for those conserved in marine cyanobacteria. They are accessible through the CyanoBase website (Nakao et al., 2010) (Table 1). Nowadays, several resources that model protein-protein interaction networks for cyanobacteria are available for researchers to examine, i.e., generic databases such as STRING, as well as specialized databases such as SynechoNET or InteroPORC. STRING covers more than 1000 organisms, containing experimentally-validated interactions, predicted and transferred interactions, together with interactions obtained through text mining (Franceschini et al., 2013). In contrast, the SynechoNET database is dedicated to *Synechocystis* 6803, and covers 2930 proteins (i.e., 79% of all predicted proteins in *Synechocystis* 6803). It includes 109,532 predicted protein-protein interactions extracted from the databases STRING (2658 proteins, 26,805 interactions), PSIMAP-based (1028 proteins, 12,748 interactions), InterDom (1760 proteins, 80,319 interactions) and iPfAM (1541 proteins, 13,448 interactions) (Kim et al., 2008). The SynechoNET visualization interface also permits the exploration of a “high confidence” sub-network composed of 509 proteins, common to all databases, connected by 1591 interactions, where each interaction has an attached score value, obtained using the arithmetic value of the scores provided by STRING and InterDom. Some of the predicted high-confidence interactions were supported by the yeast two-hybrid data in Sato et al. (2007). Another set of predicted protein-protein interactions for *Synechocystis* 6803 was obtained using a method called InteroPORC (Michaut et al., 2008). Here, interactions were computationally inferred based on orthology of proteins which are known to interact in other organisms. The protein-protein interaction network for *Synechocystis* 6803 available on the InteroPORC interface contains 2259 interactions for 807 proteins. A core of 222 interactions were supported by experimental data obtained from three curated databases IntAct (Orchard et al., 2014), MINT (Licata et al., 2012), or DIP (Salwinski et al., 2004), as well as the yeast two-hybrid data generated by Sato et al. (2007).

## Metabolomics: from pathways to whole genome fluxomics

Similar to other “omic” approaches, recent advances in HTT also have had their impact on the study of metabolites in cyanobacteria, enabling simultaneous measurement of hundreds of metabolites. The quality of these results and the breadth of metabolite coverage greatly depend on the analytical flow rate of the experimental setup. In this regard, the development of enhanced compound separation techniques, coupled to MS or nuclear magnetic resonance (NMR) spectroscopy has played a key role (Zhang et al., 2012) (Figure 1). In general, we can differentiate between three types of metabolic studies: (i) target analysis, where the goal is the identification of specific or bioactive metabolites; (ii) metabolomic profiling, where the goal is to identify as many metabolites as possible; and (iii) flux analysis, where the goal is to define fluxes through specific biochemical pathways, mainly by isotope labeling. All these approaches have been successfully applied to study metabolites in cyanobacteria and were recently reviewed in Schwarz et al. (2013).

Here, we will concentrate on approaches that deal with genome-level modeling of cyanobacteria metabolism. Such genome-scale metabolic models consider cellular metabolism in its entirety, instead of focusing on individual pathways in isolation, providing a more realistic view of the interconnection and interdependence of cellular processes. A general pre-requisite for such modeling is the existence of the whole genome sequence. In comparison to the number of available genomes, however, the number of species-specific genome-level metabolic models is still scarce (less than a 3%). This is partially due to the lack of automated modeling tools. Despite some progress in this direction (Arakawa et al., 2006; Devoid et al., 2013; Krishnakumar et al., 2013), modeling still requires intensive supervision to avoid pitfalls due to different database annotations, and there are important limitations inherent to each method currently available (Ginsburg, 2009). These limitations make modeling a cumbersome task. Nevertheless, metabolic networks are an important key to assist researchers in: the design of strains to overproduce a desired product, the identification of potential enzymes responsible for orphan reactions, the determination of optimal growth conditions to favor a reaction, the identification of coupled reaction sets, as well as evolutionary studies (Ma et al., 2013).

Once again, abundant experimental information on *Synechocystis* 6803, coupled with its use in many biotechnological applications, has supported the publication of several metabolic models for this strain (Montagud et al., 2011; Nogales et al., 2012; Knoop et al., 2013). The flow of metabolites through these models has also been determined by Flux Balance Analysis (FBA), a constraint-based method that has given rise to the new “omic” term: Fluxomics (Winter and Kromer, 2013). One advantage of FBA is that no previous knowledge of the kinetic parameters for individual metabolic reactions is required. FBA can predict the optimal steady-state fluxes required to maximize the synthesis of biomass or a product of interest, e.g., the synthesis of fatty acids in *Synechocystis* 6803.

The earliest FBA published for *Synechocystis* 6803 was carried out early in 2005 (Shastri and Morgan, 2005) and only included the evaluation of central metabolic pathways, under different light and media conditions (e.g., photoautotrophic, mixotrophic, and heterotrophic). In this study, cyclic and non-cyclic electron transport chains were considered as non-interacting events, although they share multiple components (Vermaas, 2001). This disconnection disappeared only in more recent metabolic models (Nogales et al., 2012), indicating that alternative electron flow pathways maximize growth under diverse environmental conditions, particularly when light or carbon are limiting factors. Progress has also been made in the reconstruction of central metabolic pathways. Knoop et al. (2013) were the first ones to incorporate a complete tricarboxylic acid cycle (TCA) cycle (Zhang and Bryant, 2011), while in the previous works either the glyoxylate shunt (Shastri and Morgan, 2005) or the GABA shunt were used to close the cycle (Knoop et al., 2010). Their core reconstruction encompassed 677 genes that encoded for 495 enzymes or enzyme-complexes. The annotated enzymes gave rise to 759 metabolic reactions, involving 601 metabolites. In an attempt to consolidate these networks, and at the same time to provide a tool for biologists to model their results, a user friendly-interface with a *Synechocystis*-specific section was added to web-based FAME resource (http://f-a-m-e.org/synechocystis/) (Boele et al., 2012; Maarleveld et al., 2014). The model, iTM686, is based on that of Nogales et al. (2012), but amended to include later findings, such as the complete TCA cycle (Zhang and Bryant, 2011), arginine metabolism (Schriek et al., 2009), and the proline metabolism (Knoop et al., 2010). A remarkable feature of FAME is that it permits the user to visualize flux analysis results together with gene expression data; to assist users with this objective, it has preloaded part of the CyanoEXpress microarray data.

It is important to note that these models only offer a pseudo-steady state condition, while the metabolism of cyanobacteria growing in the environment is generally under circadian control. This temporal separation is of special importance in the non-heterocyst forming nitrogen-fixing *Cyanothece* sp. ATCC 51142 (Reddy et al., 1993). A step forward in this direction was the modeling of the circadian cyclic behavior by developing separate (light/dark) biomass equations (Vu et al., 2012), adding constraints on the metabolic fluxes based on gene and protein expression (Stockel et al., 2008, 2011). A later study also incorporated constraints on the flux through alternative pathways based on ^13^C assisted metabolic flux analysis. Using the software OpenFLUX (Quek et al., 2009) the authors predicted network behavior from data obtained by the alternate use of labeled glycerol vs. unlabeled CO_2_. Rates of reactions in the carbon metabolic pathway of this *Cyanothece* strain showed that incorporation of labeled glycerol vs. unlabeled CO2 into amino acids under nitrogen-deficient and nitrogen-sufficient conditions was distinctly different. These experiments suggested that two distinct metabolic programs were active (Alagesan et al., 2013).

In some cases, the partial sequence of a genome is sufficient to build an organism metabolic network, as shown for the industrialized cyanobacterium *Spirulina (Arthrospira) platensis* (Klanchui et al., 2012). A model was derived, using a semi-automated process based on the algorithms available through the Pathway Tools software (Karp et al., 2010), and cross-comparison with data stored in the MetaCyc database (Caspi et al., 2012). This work—besides providing an exceptional model to improve the industrial use of this strain—can serve as guideline for researchers with limited programming knowledge to develop an initial metabolic draft for their organism of interest.

## Web-based tools for system biology analysis

While empowering basic and applied research in cyanobacteria, the rapidly increasing volume of data generated by HTT also provides formidable challenges to individual researchers. Data treatment is often computational intensive and needs to cover a range in raw data types, such as images of microarrays or protein gels, set sequences, and mass spectra (Figure 1). Furthermore, raw data frequently requires quality control, pre-processing, and normalization procedures prior to analysis to ensure reliability of results. To facilitate the use of available data for scientists, several web-based or community resources have been established specifically for cyanobacteria. They allow users to custom analyze data repositories as part of their experimental design protocol, and to compare their data with other research results (Table 2).

**Table 2:** **Web-based analytical tools with a specific focus on cyanobacteria as at April 2014**.

**Website**	**In-built data**	**Analysis tools**	**Visualization**	**Other advantages**
CyanoBase	Genetic	Blast2, KazusaMart	YES (Genome view)	Downloadable data
CyanoBIKE	Genetic transcriptomic metabolic	In-built and custom tools	NO	Programmable
CyanoEXpress	Transcriptomic	Co-regulation and cluster analysis	YES (GeneXplorer)	Special datasets
Cyanorak	Genetic	Blast	NO	Downloadable data
ProPortal	Genetic transcriptomic ecologic	Blast, cluster, enrichment analysis	NO	Species-specific data
CINPER	Genetic data from biosubsystems	Network mapping, enrichment analysis	YES	Exports networks, user input
FAME	Metabolic networks	FBA Gene expression Image manipulation	YES	Export images and networks

The central and most widely-used resource in the field is CyanoBase (http://genome.microbedb.jp/cyanobase/; Nakao et al., 2010). Started in 1995, this database includes currently sequenced and annotated genomes for 39 species of cyanobacteria. Although it contains only a very limited number of analysis tools (i.e., Blast2 for genes and genomes similarities searches, and KazusaMart to quickly convert between IDs in different formats, powered by the free software BioMart), it allows the user to explore genomes, as well as to obtain gene annotations. For instance, after querying for a gene, the user can obtain information on relevant publications, number of predicted transmembrane regions, putative protein-protein interactions, orthologous genes in other cyanobacteria, and more importantly download sequence data. The current version of CyanoBase was updated by the Kazusa DNA Research Institute (Nakao et al., 2010) to include a full text search, gene indexing, and mutants. It is currently maintained by Nakamura's Laboratory at the National Institute of Genetics.

The Virginia Commonwealth University hosts the BioBike Server for the Public Cyanobacterial Edition v5.2. CyanoBike (http://biobike-8003.csbc.vcu.edu/biologin) is a web-based, programmable knowledge base, designed to make genomic, metabolic, and experimental data available to the public. It has built-in tools to manipulate and analyze these data (Massar et al., 2005; Elhai et al., 2009), although they require some basic programming skills for their use. Researchers can select databases devoted to marine cyanobacteria or to other specific organisms. Drop down menus are available to select functions, e.g., to compare proteins or gene-strings. It currently has 13 fully sequenced cyanobacterial strains in its dataset, and expects to include another 20 over the next year. Twelve microarray datasets also are integrated, including nine for *Synechocystis* PCC 6803 (corresponding to normalized data available through the KEGG EXPRESSION Database). It is updated regularly and is funded by the National Science Foundation (USA).

CyanoEXpress is a web-served dedicated to transcriptomics data for cyanobacteria (http://cyanoexpress.sysbiolab.eu/; Hernandez-Prieto and Futschik, 2012). Currently, it includes expression data only for *Synechocystis* PCC 6803, with 718 microarray measurements compiled from 33 independent studies with both environmental and genetic perturbations. As such, there are 177 expression entries for 3073 genes. It aims to assist researchers in the characterization and functional annotation of genes using the guilty-by-association principle. Its visualization tool is a modified version of GeneXplorer (Rees et al., 2004). Different data subsets can be selected, with genes associated to different functions, based on whether all perturbations (genetic and environmental) or only environmental ones are included. CyanoEXpress is regularly updated with new data retrieved from the public repositories: GEO, ArrayExpress, and KEGG. Future versions will also include expression data for other cyanobacteria, such as *Prochlorococcus* and *Synechococcus*.

Cyanorak (http://www.sb-roscoff.fr/cyanorak) is a dedicated resource for curation and annotation of clusters of orthologous sequences from marine picocyanobacteria. Cyanorak v.1 contains three *Prochlorococcus* and 11 *Synechococcus* genomes (Dufresne et al., 2008); Cyanorak v.2 containing 14 *Prochlorococcus*, 3 *Cyanobium*, and 40 *Synechococcus* genomes will soon be released. The current version allows the user to export individual Genbank files, as well as protein/gene sequences as FASTA files. More importantly, Cyanorak is manually curated, providing an updated version of the genomes available. The curation results in detailed descriptions for many clusters of gene orthologs, which can be retrieved using a search engine with different options (fast/advanced) set by the user.

ProPortal (http://proportal.mit.edu/) aims to provide easy access to the growing genetic database devoted to the cyanobacterium *Prochlorococcus* and its phages; to facilitate its use as a model system for systems biology (Kelly et al., 2012). The database includes genomes of cultured isolates of *Prochlorococcus* and their phages (and 11 strains of *Synechococcus*), as well as processed expression data from microarray experiments. Users can search for orthologous gene clusters, compare genomes from different populations, and identify up- or down-regulated genes under different environmental stressors (light, nitrogen, phosphate and iron) using various modules. The ProPortal database (Version 3) was recently updated with new gene cluster definitions (Kelly et al., 2013), so that it now contains 68 (24 host, 44 phage) genomes and 55,622 genes.

CINPER (http://csbl.bmb.uga.edu/cinper/) is one of the newer websites devoted to prokaryotes, developed by the Computations Systems Biology Laboratory at the University of Georgia. It focuses on networks, mapping well-known genes from multiple template genomes to a target genome and has been applied to study osmoregulation in *Synechococcus* sp. WH8102 (Mao et al., 2012). Networks can be validated with gene expression data (provided by the user), and can be visualized and explored using the Cytoscape Web interface (Lopes et al., 2010). One of its main advantages is that networks can be exported as images in various formats or as xml-based formats, i.e., svg and xgmml.

Finally, FAME (http://f-a-m-e.org/synechocystis/), previously discussed in the metabolomics section, provides the user with the option to download editor-friendly image files of the *Synechocystis* 6803 metabolic network. This model can be used as a framework to modify and build other organism-specific networks.

## Case study: inferring conserved regulatory interactions from inter-species gene expression data

The identification of conserved elements in regulatory networks is a suitable task to demonstrate some of the capabilities of the above web-resources. For this, we compare two time-series of expression data under iron limitation for *Prochlorococcus* MED4 (Thompson et al., 2011) (available through the ProPortal database) and *Synechocystis* 6803 (Hernandez-Prieto et al., 2012) (available through the GEO database). Here, we assume if a gene is present in two given organisms, then its function also is likely to be conserved. Our first step is therefore to identify putative orthologous genes between these cyanobacteria, using the tools provided in the IMG database (https://img.jgi.doe.gov/) to compare genomes, under the option “Genome Gene Best Homologs” (Table S2). After selecting the genomes to compare, and deciding on a minimum threshold for identification, this option generates a tabulated file with the gene IDs from the reference genome, and their corresponding homologs for the given organism. In our case, to maximize the number of identified homologs, we use the lowest identity-percent allowed (20%). The output file had a total of 1050 unique *Prochlorococcus* MED4 genes (59.8% of the total genes) with identified homologs in *Synechocystis* 6803. This straight-forward step results in a list of *Synechocystis* 6803 and *Prochlorococcus* MED4 genes, annotated in a different format (i.e., SYNGTS_0535; PMM1032) to that used in the microarray annotation (i.e., slr2043; PMED4_11751), following the CyanoBase and ProPortal nomenclature, respectively. In order to merge both nomenclatures, we use equivalent identifiers provided for each genome through the databases: UniProt (http://www.uniprot.org/), CYORF (http://cyano.genome.jp/), and NCBI. A key conversion table is available in the supplementary material (Table S2).

**Figure 5:**
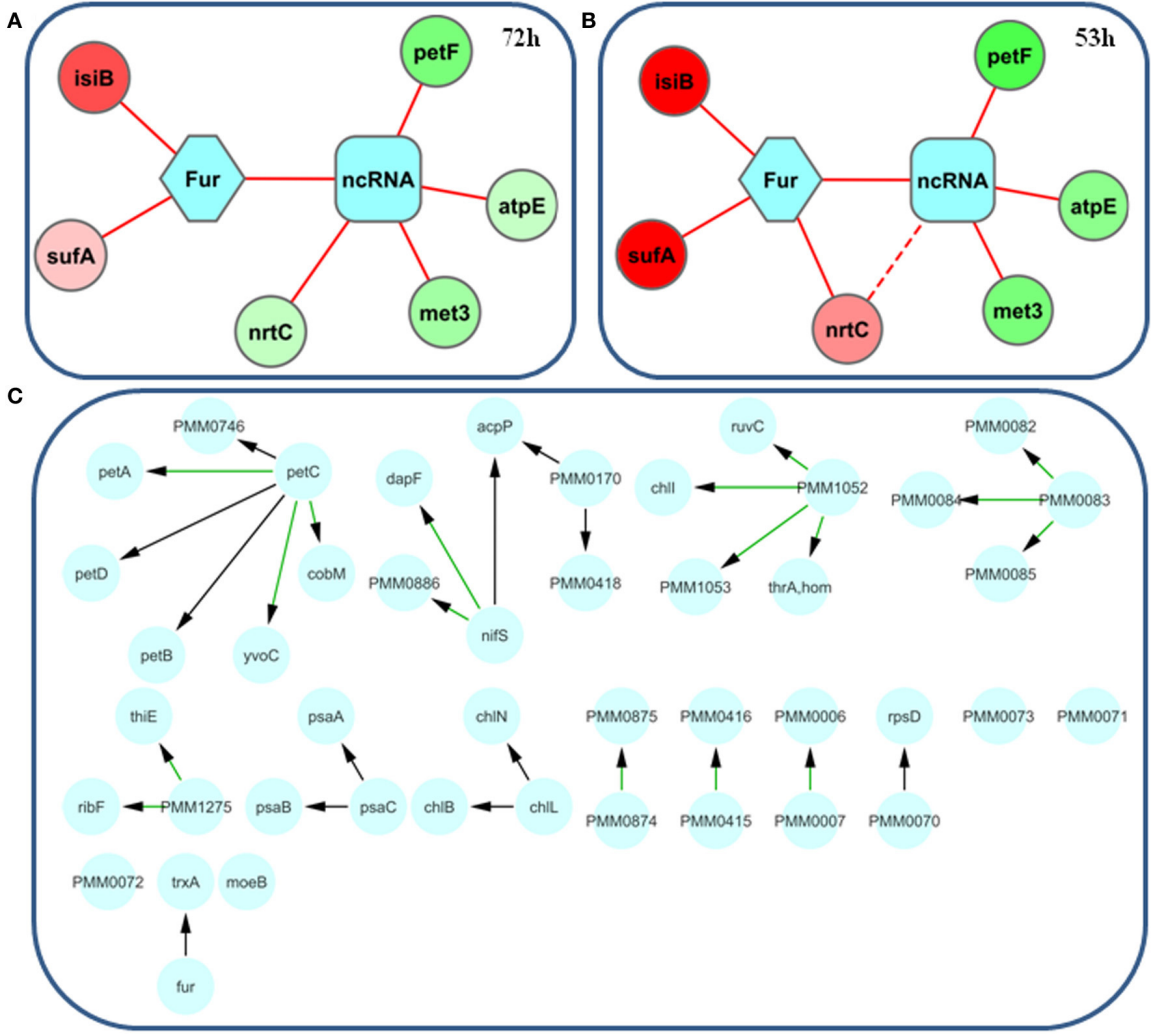
**Iron regulatory networks based on the differential expression upon iron limitation of genes conserved (identity larger than 20%) between *Synechocystis* sp. PCC6803 and *Prochlorococcus* MED4**. The ferric uptake regulator (Fur) and a hypothetical ncRNA were set as central regulatory elements (in blue) following the well-described iron regulatory network of *E. coli*. Circular nodes were colored using a gradient from green to red, reflecting differential expression upon iron depletion at 72 h for *Synechocystis* 6803 **(A)** and 53 h for *Prochlorococcus* MED4 **(B)**. Red edges indicate a putative repression by the regulatory element. The apparent differential regulation of *cmpA/nrtC* is discussed in more detail in the case study. **(C)** CINPER-generated network created using the keyword set to Fur, with *Synechocystis* 6803 as the reference organism, and *Prochlorococcus* MED4 as the target.

Once differences in annotation have been resolved, we can compare differentially-expressed genes upon iron limitation in *Prochlorococcus* MED4, using the criteria provided in the corresponding publication (Thompson et al., 2011). In the case of *Synechocystis* 6803, we limit the list of genes to those in which expression was highly correlated (*r*_*s*_ ≥ 0.8) with that of the iron-stress induced A and B (*isiA* and *isiB*) genes, known to be regulated by the ferric uptake regulator (Fur) (Kunert et al., 2003). Surprisingly, of these 97 genes in *Synechocystis* 6803, only 6 have homologs (based on at least a 20% homology) in the 74 differentially-expressed genes in *Prochlorococcus* MED4. To facilitate the visualization of these results, we built a small network with the identified genes connected to Fur, the main transcription factor involved in iron adaptation for *Synechocystis* and *Prochlorococcus* (Figures 5A,B). Fur homologs are present in both organisms (sll0567 in *Synechocystis* 6803 and PMM0637 in *Prochlorococcus* MED4). In the presence of sufficient iron, Fur binds upstream of genes involved in iron acquisition, blocking their transcription. In iron-limited conditions, Fur loses its affinity to the promoter regions, permitting the binding of specific sigma factors, and thus, transcription of downstream genes. In *E. coli* a second level of regulation is exerted by the ncRNA RyhB (Masse and Gottesman, 2002), which is under the control of Fur in iron-limited conditions. Upon transcription, RyhB promotes degradation of mRNAs, encoding non-essential iron-using proteins during iron limitation (Massé et al., 2003). Similar ncRNAs induced under iron-limiting conditions were identified in *Synechocystis* 6803 (Hernandez-Prieto et al., 2012), and candidates also exist in *Prochlorococcus* (Steglich et al., 2008). Therefore, to complete the network, we connect the genes repressed by iron, to this hypothetical regulatory element (ncRNA), even though this connection is not yet supported by experimental data. Thus, the generated regulatory networks are only hypothetical. Finally, we visualize the networks using the Cytoscape environment (Shannon et al., 2003); connections between genes and regulatory elements were established based on whether expression was higher or lower under iron-limiting conditions. Inspection of the two network shows that the direction of expression changes is conserved in both cyanobacteria upon iron limitation; only one discrepancy associated with CmpA exists (a subunit of the bicarbonate transporter). This discrepancy appears to be related to the homology of the gene product of PMM0370 (annotated as putative cyanate ABC transporter) to two different transporter subunits, NrtC and CmpA. To resolve this discrepancy, we specifically looked for homologous genes to PMM0370, using the local blastp tool in the NCBI database. The top scoring protein in this case, was not CmpA, but NrtC (sll1452, a subunit of the nitrate transporter), which is also up-regulated in *Synechocystis* 6803 (Hernandez-Prieto et al., 2012). In fact, PMM0370 is part of the cynABDS operon and displays a common expression response to nitrogen limitation together with co-localized genes (Kamennaya and Post, 2011). To compare our regulatory network based on conservation of gene expression, with an automatically generated network, we created one with the keyword set to Fur, using CINPER with *Synechocystis* 6803 as the reference organism, and *Prochlorococcus* MED4 as the target (Figure 5C). The predicted protein-protein interactions related to the keyword were extracted from different sources, e.g., SEED, and the STRING database (Mao et al., 2012). In the CINPER network, Fur is identified with two *Synechocystis* 6803 proteins, FurA (sll0567) and PerR (slr1738), both related to Fur in *E. coli*. Of these two proteins, only FurA, is involved in iron regulation; while PerR is related to gene control during redox stress (Li et al., 2004; Shcolnick et al., 2009).

This illustrative example, using available online tools and organism-specific data, shows how information from well-studied organisms can be used to interpret results obtained for phylogenetically-related organisms.

## Conclusions

In this review, we have illustrated the breadth and depth of currently available “omic” data in public repositories. In addition, we have emphasized the value and advantages of these types of data for systems-level analyses, and the novel research that can result from integrating species-specific data into a wider context. Various examples were given for systems-level analysis of marine cyanobacteria, including the identification of class-specific genes located in genomic islands in *Prochlorococcus* using metagenomics, development of regulatory networks for nitrogen metabolism in *Synechococcus* through transcriptomics, the effects of phosphate limitation on *Synechococcus* in proteomics, and identification of two distinct metabolic pathways for different nitrogen conditions in *Cyanothece* in metabolomics. These results illustrate the power and potential of systems-level approaches in biological research.

In addition, we provided an overview of currently available user-friendly tools for researchers to use to manipulate “omic” data for marine cyanobacteria, illustrating their scope with a case study. The case study emphasizes both the types of networks that can be generated from online data, as well as their dependence on data quality. It also highlights some of the present limitations in automatically-generated networks, suggesting that there is still a vital role to supervised curation of data analysis.

At present, the number of researchers with the bioinformatic skills is growing, in response to the need to deal with data generated by HTT. This enhanced-skills base will continue to improve our capacity to connect physiological aspects with “omic” data, and to develop better bioinformatics tools to process large complex data sets. It is these system-level approaches that will support the future of meta-studies, and allow us to offset the quantity of HTT data with quality biological interpretations. New insights gained from system-level approaches will further research in both science and industry related to cyanobacteria.

### Conflict of interest statement

The authors declare that the research was conducted in the absence of any commercial or financial relationships that could be construed as a potential conflict of interest.
